# Management of antipsychotic-induced tardive dyskinesia with calcium channel blockers and botulinum toxin: a case report

**DOI:** 10.1192/j.eurpsy.2025.2270

**Published:** 2025-08-26

**Authors:** A. Souidi, S. Belbachir, A. Ouanass

**Affiliations:** 1 Psychiatry B, Ar-razi hospital, Salé, Morocco

## Abstract

**Introduction:**

Tardive dyskinesia (TD) is an iatrogenic disorder characterized by a range of movement abnormalities, described as irregular, stereotyped, and choreiform caused by exposure to antipsychotics. This condition can lead to significant or disabling symptoms that affect quality of life. However, the exact mechanism underlying TD remains unclear. Pharmacotherapy of TD includes cholinergic drugs, benzodiazepines, antioxidants, amantadine, propanolol, botulinum toxin, whereas the non-pharmacotherapy approach includes modified electroconvulsive therapy and deep brain stimulation. We successfully treated a chronic schizophrenia patient with comorbid TD using Aripiprazole and botulinum toxin after trying calcium channel blockers in association with Aripiprazole and vitamin E.

**Objectives:**

To determine the effects of calcium channel blocker drugs (Amlodipine) for treatment of neuroleptic-induced tardive dyskinesia in people with schizophrenia, and the efficiency of botulinum toxin in treating induced tardive dyskinesia.

**Methods:**

Through a case report and a literature review, our work aims to study antipsychotics induced tardive dyskinesia and its pharmacotherapy especially the use of calcium channel blocker and botulinum toxin in association with Aripiprazole.

**Results:**

A 22 years male, diagnosed with schizophrenia since the age of 16 years, with one hospitalization. The evolution of his disease was marked by the development of a tardive dyskinesia, cervical and brachial movements disorders with rapid worsening in few months, the patient was treated with olanzapine oral administration. During his follows-up, his tardive dyskinesia disn’t resolve despite switching the incriminated antipsychotic to Aripiprazole, lowering the dose since he was on complete remission. we added benzodiazepines and vitamin E to his treatment, before trying the calcium channel blocker for two months, with no improvement, on the contrary a worsening was noted using the Unified Dyskinesia Rating Scale. The aggravation of the movements was a reason for the patient attempted suicide, after this late incident we chose to try the botulinum toxin injections. His tardive dyskinesia was spectacularly improved within the fisrt injections.

**Conclusions:**

Antipsychotic drugs are known to cause a variety of adverse effects, including tardive dyskinesia. Hence the importance of knowing the pharmacotherapy and non-pharmaco- therapy to manage this effect, through this case report where it tardive dyskinesia got treated after using botilinum toxin injections with a spectacular improvement in its Unified Dyskinesia Rating Scale. In our case, we had no response after adding calcium channel blocker .The effects of calcium channel blockers for antipsychotic-induced tardive dyskinesia are unknown, and it use is still limited.

**Disclosure of Interest:**

None Declared

